# Editorial: Cross-Domain Analysis for “All of Us” Precision Medicine

**DOI:** 10.3389/fgene.2021.713771

**Published:** 2021-07-01

**Authors:** Tao Zeng, Tao Huang, Chuan Lu

**Affiliations:** ^1^CAS Key Laboratory of Computational Biology, Bio-Med Big Data Center, Shanghai Institute of Nutrition and Health, University of Chinese Academy of Sciences, Chinese Academy of Sciences, Shanghai, China; ^2^Shanghai Institute of Nutrition and Health, University of Chinese Academy of Sciences, Chinese Academy of Sciences, Shanghai, China; ^3^Department of Computer Science, Aberystwyth University, Aberystwyth, United Kingdom

**Keywords:** cross-domain, precision medicine, artificial intelligence, machine learning, omics

Precision medicine aims to combat complex human diseases by providing preventative, diagnostic, and treatment tools for each patient rather than each cohort. Along with the development of precision medicine (formerly called personalized medicine) (Aronson and Rehm, 2015), the recent “All of Us” initiative seeks to gather data related to “lifestyle, environment, and biology” from a wide range of people in order to better understand individual differences and improve precision medicine (All of Us Research Program Investigators et al., 2019). “All of Us” precision medicine is seeking easy, low-cost, non-invasive, and effective early clinical detection approaches, which include novel biological technology (BT) and information technology (IT) in precision healthcare research. Importantly, many types/domains of data from new technologies can be utilized, such as voice/text recording, image scanning, behavioral monitoring, and feature phenotyping, in addition to omics data from high-throughput technologies (Zeng, 2021). These consist of cross-domain data, where the micro cross-domain can cover conventional omics data and the macro cross-domain can bridge the omics with other data types. Therefore, the cross-domain analysis is a key to understanding “All of Us” precision medicine.

In particular, the artificial intelligence (AI) model and method (e.g., deep learning) has achieved great success in analyzing image data in many fields and is bringing new opportunities to clinical research (Zeng et al., 2021). However, for cross-domain analysis in “All of Us” precision medicine, several challenges still exist: the numbers of variables in different domains are unbalanced, and the bias coming from the dominant domain should be tackled (Zhang et al., 2020); the number of observations is small or not large enough in clinical or biological fields, thus small-sample modeling is required (Lai et al., 2020); the samples in different domains may be not matched, so that heterogeneous integration should be considered (Yang et al., 2020); and many data organizations exist, so data without a vector or matrix form needs a reasonable and powerful transformation/representation (Wang et al., 2021).

This Research Topic serves as an update of our previous topic (Zeng et al., 2019), “Machine Learning Advanced Dynamic Omics Data Analysis for Precision Medicine,” with a focus on the application of artificial intelligence with cross-domain data for further developing “All of Us” precision medicine.

At present, we do not have a clear understanding of the molecular pathogenesis of patent ductus arteriosus (PDA). Chen et al. screened the rare copy number variants of 39 PDA patients and 100 healthy children by whole exome sequencing. This study was narrowed down by network and enrichment based analysis, and finally selected 7 candidate genes significantly expressed in human embryonic heart, which include CHRNA4 as potential genetic cause of PDA.

Yan et al. investigated the potential of serum miRNAs as signatures for pancreatic cancer (PC). First, they analyzed the expression differences of miRNAs between PC patients and healthy controls by feature selection approaches, obtaining a group of PC signatures. Next they compared miRNAs' expressions between inoperable and operable PC patients, identifying lots of operability miRNAs. These findings revealed a non-invasive way on the diagnosis and therapy of pancreatic cancer.

Xu et al. applied network-based bioinformatics analysis for reanalyzing temporal gene expression data related to Infantile hemangioma (IH), with the purpose of distinguishing genes for proliferative and regressive IH lesions. This analysis pipeline included Short Time-series Expression Miner, weighted gene co-expression network analysis, and area under curve ranking, which determined key hub genes which were verified using qRTPCR.

Restoration of altered transcriptional regulations in disease states should be an important action for the pathogen genes associated with disease diagnosis and prognosis (Lu et al., 2020). The quantitative relation between transcriptional regulation and disease phenotypes can help detect such targeted genes for transcriptional modification. Duan et al. proposed a model-based quantitative relationship measurement between mRNA (non-TF) genes and Transcription Factor (TF) genes. They further applied this method to detect quantitatively altered mRNA genes in lung cancer on the basis of one learning dataset and several independent evaluation datasets, which provided new candidates for lung cancer diagnosis, prognosis, and treatment.

Complex diseases are determined by many heterogeneous biological factors (Su et al., 2020), thus, omics integration analysis should help capture new prognostic signatures and models for disease prognosis. Ren et al. explored the key biomarkers of early-stage Lung adenocarcinoma and built a prognostic model on the combination of gene transcription and methylation. In the re-analysis of public RNA-seq and methylation microarray data, with a series of statistical, network-based and machine learning approaches, a prognostic index based on 17 methylation driver genes, and 4 CD8 T cell-related genes was established for predicting the survival risk of lung cancer patients in early-stage.

Finally, we sincerely thank the reviewers; their great efforts ensure the high quality of all articles in this topic. This Research Topic should bring more attention to the important topics of “All of Us” precision medicine based on machine learning and cross-domain data.

## Author Contributions

TZ drafted the manuscript. TZ, TH, and CL revised the manuscript. All authors contributed to the article and approved the submitted version.

## Conflict of Interest

The authors declare that the research was conducted in the absence of any commercial or financial relationships that could be construed as a potential conflict of interest.

## References

[B1] All of Us Research Program Investigators Denny J. C. Rutter J. L. Goldstein D. B. Philippakis A. Smoller J. W. Jenkins G. (2019). The “All of Us” research program. N. Engl. J. Med. 381, 668–676. 10.1056/NEJMsr1809937 31412182 PMC8291101

[B2] Aronson S. J. Rehm H. L. (2015). Building the foundation for genomics in precision medicine. Nature 526, 336–342. 10.1038/nature15816 26469044 PMC5669797

[B3] Lai N. Kan M. Han C. Song X. Shan S. (2020). Learning to learn adaptive classifier-predictor for few-shot learning. IEEE Trans. Neural. Netw. Learn. Syst. 10.1109/TNNLS.2020.3011526. [Epub ahead of print]. 32755872

[B4] Lu Y. Xue J. Deng T. Zhou X. Yu K. Deng L.. (2020). Safety and feasibility of CRISPR-edited T cells in patients with refractory non-small-cell lung cancer. Nat. Med. 26, 732–740. 10.1038/s41591-020-0840-5 32341578

[B5] Su Y. Chen D. Yuan D. Lausted C. Choi J. Dai C. L.. (2020). Multi-omics resolves a sharp disease-state shift between mild and moderate COVID-19. Cell 183, 1479–1495 e1420. 10.1016/j.cell.2020.10.037 33171100 PMC7598382

[B6] Wang J. Ma A. Chang Y. Gong J. Jiang Y. Qi R.. (2021). scGNN is a novel graph neural network framework for single-cell RNA-Seq analyses. Nat. Commun. 12:1882. 10.1038/s41467-021-22197-x 33767197 PMC7994447

[B7] Yang H. Cao H. He T. Wang T. Cui Y. (2020). Multilevel heterogeneous omics data integration with kernel fusion. Brief Bioinform. 21, 156–170. 10.1093/bib/bby115 30496340

[B8] Zeng T. (2021). Repurpose analysis expanding biomedical benefits by omics data integration, in Systems Medicine, ed. Wolkenhauer O. (Oxford: Academic Press) 94–102. 10.1016/B978-0-12-801238-3.11387-X

[B9] Zeng T. Huang T. Lu C. (2019). Editorial: Machine learning advanced dynamic omics data analysis for precision medicine. Front. Genet. 10:1343. 10.3389/fgene.2019.01343 32117409 PMC7010801

[B10] Zeng T. Yu X. Chen Z. (2021). Applying artificial intelligence in the microbiome for gastrointestinal diseases: a review. J. Gastroenterol. Hepatol. 36, 832–840. 10.1111/jgh.15503 33880762

[B11] Zhang M. L. Li Y. K. Yang H. Liu X. Y. (2020). Towards class-imbalance aware multi-label learning. IEEE Trans. Cybern. 10.1109/TCYB.2020.3027509. [Epub ahead of print]. 33206614

